# The effect and mechanism of cypermethrin-induced hippocampal neurotoxicity as determined by network pharmacology analysis and experimental validation

**DOI:** 10.1080/21655979.2021.2000106

**Published:** 2021-11-22

**Authors:** Jianan Li, Haoran Bi

**Affiliations:** a KeyLaboratory of Environment and Health, College of Public Health, Xuzhou Medical University, Xuzhou, China; b Department of Biostatistics, College of Public Health, Xuzhou Medical University, Xuzhou, China

**Keywords:** Network pharmacology, experimental validation, Cypermethrin, hippocampal neurotoxicity

## Abstract

Cypermethrin (CMN) is a widely used artificial synthetic pesticide that causes neurotoxicity in the hippocampus. However, the underlying toxicological targets and mechanisms remain unclear. In this study, network pharmacology analysis and *in vitro* models were integrated to investigate the effect and mechanism of CMN-induced hippocampal neurotoxicity. A total of 88 targets of CMN-induced hippocampal neurotoxicity were predicted. Gene Ontology (GO) and Kyoto Encyclopedia of Genes and Genomes enrichment (KEGG) analyses suggested that these targets were related to multiple GO terms and signaling pathways. To further investigate underlying mechanism, the top 10 hub targets (Akt1, Tnf, Ptgs2, Casp3, Igf1, Sirt1, Jun, Cat, Il10, and Bcl2l1) were screened. Furthermore, cell viability and lactate dehydrogenase (LDH) assays demonstrated that CMN was toxic to HT22 cells in a time- and dose-dependent manner. Terminal deoxynucleotidyl transferase dUTP nick end labeling (TUNEL) staining revealed that treatment with CMN increased the proportion of apoptotic cells. In addition, the real-time quantitative polymerase chain reaction (RT-qPCR) results indicated that CMN altered the mRNA expression levels of most of the hub targets, with the exceptions of Igf1 and Jun. The results demonstrated that multiple targets and signaling pathways were involved in CMN-induced hippocampal neurotoxicity. These findings provided reference values for subsequent studies of the toxicological mechanism of CMN.

## Introduction

In agriculture, pesticides can improve crop yield and quality by killing or controlling insects, weeds, and detrimental microorganisms [1]. Pyrethroids are a large family of synthetic insecticides with chemical structures similar to that of the natural pesticide pyrethrum, which is produced by the chrysanthemum flower [2]. Cypermethrin (CMN, [cyano-(3-phenoxyphenyl)methyl] 3-(2,2-dichloroethenyl)-2,2-dimethylcyclopropane-1-carboxylate, PubChem CID: 2912) is a type II synthetic pyrethroid that is extensively used in residential, industrial, and agriculture applications [3]. Due to such widespread use, the potential adverse effects of CMN residues in biological and environmental matrices have led to global concerns about the effects of CMN on human health.

CMN is toxic to the reproductive organs, liver, and neurons of various species [4–6]. Hence, the neurotoxicity of CMN continues to receive an increasing amount of attention. Upon breaching the blood-brain barrier, CMN is reported to cause neuronal injury and subsequent motor and cognitive deficits in rodents [7]. Cognitive injury is an established marker of aging and neurodegenerative disease, especially to the hippocampus [8]. Several studies have reported that CMN caused injury to the hippocampal neurons and further impaired cognitive functions, although the underlying molecular mechanisms remain unclear [6,9,10]. CMN is considered a multi-target toxicant that exploits a range of mechanisms via chloride channels, oxidative stress, and ATPases [11]. Therefore, it is essential to investigate the neurotoxic mechanisms of CMN in the hippocampus systematically.

Following the development of network database retrieval, systems biology, bioinformatics, and computer prediction technologies, network pharmacology has become a novel tool to comprehensively elucidate the relationships between chemicals and specific phenotypes [12]. The core idea of network pharmacology is to construct a ‘chemical-target-disease’ interaction model [13]. Recent studies have clarified the toxicological mechanisms of a series of chemicals, such as oxalic acid, cantharidin, and esculentoside A, with the use of network pharmacology [14–16].

The combination of network pharmacology and experimental validation has emerged as a preferred strategy to explore the core targets and mechanisms of chemicals [17–19]. In the present study, network pharmacology was used to predict the neurotoxic mechanisms of CMN by screening multiple candidate targets, predicting possible signaling pathways, calculating the top 10 hub targets, and constructing a CMN-targets-hippocampal neurotoxicity network. Furthermore, an *in vitro* exposure model was established to verify the neurotoxic effect of CMN, including cell proliferation, cytomembrane damage, and apoptosis. Finally, changes to the expression profiles of key targets were detected. The results of this study will help to clarify the neurotoxic mechanisms of CMN.

## Materials and methods

### Prediction of targets related to CMN

The SwissTargetPrediction database (http://www.swisstargetprediction.ch) [14], Comparative Toxicogenomics Database (CTD, http://ctdbase.org) [16], and STITCH database (http://stitch.embl.de) [20] were selected to predict potential targets of CMN. The three-dimensional (3D) structure of CMN was retrieved from the PubChem database (https://pubchem.ncbi.nlm.nih.gov) [14] and uploaded to the above three databases. For the SwissTargetPrediction and CTD databases, *Mus musculus* (for further validation of the results *in vitro*) was selected as the target species and all other variables were set at default values. For the STITCH database, *M. musculus* was selected as the target species, the minimum required interaction score was set at 0.4, and all other variables were set at default values. Standard gene names were retrieved from the UniProt database (http://www.uniprot.org) [13].

### Prediction of targets related to hippocampal neurotoxicity

The CTD and National Center for Biotechnology Information (NCBI) gene databases (https://www.ncbi.nlm.nih.gov/gene) [21] were used to predict the potential targets of neurotoxicity of the *M. musculus* hippocampus with the keywords ‘hippocampal neurotoxicity,’ ‘hippocampal toxicity,’ ‘hippocampal injury,’ and ‘hippocampal damage.’ All target names were normalized as described above.

### Venn analysis and construction of a protein–protein interaction (PPI) network

The chemical and phenotype targets obtained above were uploaded into the Draw Venn Diagram tool (http://bioinformatics.psb.ugent.be/webtools/Venn) [16]. The intersectional genes were considered as the targets of CMN-induced hippocampal neurotoxicity. A PPI network of *M. musculus* was constructed based on the STRING database (http://string-db.org) [13] with a minimum required interaction score of 0.4 and visualized using Cytoscape 3.6.0 software (http://chianti.ucsd.edu/cytoscape-3.6.0) [14].

### Gene Ontology (GO) enrichment analysis and Kyoto Encyclopedia of Genes and Genomes (KEGG) pathways

The Metascape database (https://metascape.org) [22] was used for GO enrichment analysis and KEGG pathway identification in *M. musculus*. GO terms with a probability (*p*) value of < 0.05 were considered significantly enriched. The top 10 GO terms in the biological process (BP), cellular component (CC), and molecular functions (MF) domains, as well as the top 20 KEGG pathways, were used for further analysis.

### Calculation of hub targets and construction of the CMN-targets-hippocampal neurotoxicity network

For further experimental validation, the maximal clique centrality (MCC) method of the Cytoscape plugin cytoHubba was selected to identify the top 10 hub targets. The chemical, targets, and phenotype were inputted into Cytoscape 3.6.0 software to construct a CMN-targets-hippocampal neurotoxicity network. The nodes were scored and ranked according to network features with the use of the Network Analyzer tool.

### Cell culture and treatment

Mouse hippocampal neuronal HT22 cells were purchased from Shanghai Guandao Biological Engineering Co., Ltd. (Shanghai, China) and cultured in high-glucose Dulbecco’s modified Eagle’s medium (HyClone Laboratories, Inc., South Logan, UT, USA) supplemented with 10% fetal bovine serum (ScienCell Research Laboratories, Inc., Carlsbad, CA, USA) and 1% double antibodies (HyClone Laboratories, Inc.) under a humidified atmosphere of 5% CO_2_/95% air at 37°C until reaching approximately 80% confluence. CMN (98% pure) was obtained from Shanghai Aladdin Biochemical Technology Co., Ltd. (Shanghai, China) and dissolved in dimethyl sulfoxide (DMSO) (MP Biomedicals, Solon, OH, USA) to concentrations of 0, 50, 100, 200, 400, and 800 µM with a final DMSO concentration of 0.1% (0 µM) as vehicle control.

### Cell viability assay

Cell viability was assessed using a Cell Counting Kit-8 (CCK8) (Biosharp, Hefei, China) in accordance with the manufacturer’s instructions. Briefly, 5 × 10^3^ HT22 cells were seeded into the wells of 96-well plates and treated with 0, 50, 100, 200, 400, and 800 µM CMN for 24, 48, or 72 h, respectively. Afterward, 10 μL of CCK8 reagent were added to each well. After 1.5 h of incubation at 37°C, the absorbance was detected at 450 nm. The results of five independent experiments were averaged for analysis.

### Lactate dehydrogenase (LDH) assay

Cytotoxicity was monitored using an LDH Release Assay Kit (Beyotime Institute of Biotechnology, Shanghai, China) in accordance with the manufacturer’s instructions. Briefly, 5 × 10^3^ HT22 cells were seeded into the wells of 96-well plates and treated with 0, 100, 200, and 400 µM CMN for 72 h. The medium was centrifuged and 120 μL of the supernatant was transferred into the wells of new 96-well plates. Then, 60 μL of LDH detection reagent were added to each well, and the plated was incubated for 30 min in the dark. Afterward, absorbance was detected at 490 nm. The results of three independent experiments were averaged for analysis.

### Terminal deoxynucleotidyl transferase dUTP nick end labeling (TUNEL) assay

Apoptosis of HT22 cells was measured using a One-step TUNEL Apoptosis Assay Kit (Beyotime Institute of Biotechnology) in accordance with the manufacturer’s instructions. Briefly, 3 × 10^4^ HT22 cells were seeded into the wells of 6-well plates and treated with 0, 100, 200, and 400 µM CMN for 72 h. Afterward, the cells were fixed with 4% paraformaldehyde for 30 min, permeabilized with 0.1% Triton X-100 for 5 min, and then incubated with 100 μL of TUNEL detection reagent for 1 h in the dark. An anti-fluorescence quencher with 4ʹ,6-diamidino-2-phenylindole was used for sealing. Finally, the stained cells were examined under an inverted fluorescence microscope.

### RNA extraction and real-time quantitative polymerase chain reaction (RT-qPCR)

Total RNA was isolated from HT22 cells treated with 0, 100, 200, and 400 µM CMN for 72 h using TRIzol reagent (Thermo Fisher Scientific, Waltham, MA, USA) and synthesized into cDNA using the Prime Script 1st strand cDNA Synthesis Kit (Takara Bio, Inc., Kusatsu, Shiga Prefecture, Japan) in accordance with the manufacturer’s instructions. The primer sequences were designed and synthesized by Shanghai Generay Biotech Co., Ltd (Shanghai, China) and listed in Table S1. The cDNA was amplified using the SYBR® Premix Ex TaqTM II (TaKaRa Bio Inc.) in ABI 7500 Real-Time PCR system with a normal reaction condition as described in our previous study [23]. Relative mRNA expression was analyzed via normalized to β-actin and calculated using the 2^−ΔΔCT^ method.

### Statistical analysis

All statistical analyses were performed using IBM SPSS Statistics for Windows, version 20.0. (IBM Corporation, Armonk, NY, USA) and the results are presented as the mean ± standard error of the mean (SEM). Two-way analysis of variance (ANOVA) was performed to analyze the cell viability assay data, while one-way ANOVA was used to analyze all other data. Dunnett’s test was used for comparisons of multiple groups. A *p* value of < 0.05 was considered statistically significant.

## Results

The molecular mechanisms underlying CMN-induced hippocampal neurotoxicity remain poorly characterized. In this study, network pharmacology was used to predict the targets and potential pathways of CMN to trigger hippocampal neurotoxicity. In addition, HT22 cells were applied as an *in vitro* exposure model to validate the neurotoxic effect of CMN and changes to expression profiles of hub targets.

### Candidate targets of CMN-induced hippocampal neurotoxicity and PPI network construction

**Figure 1.**
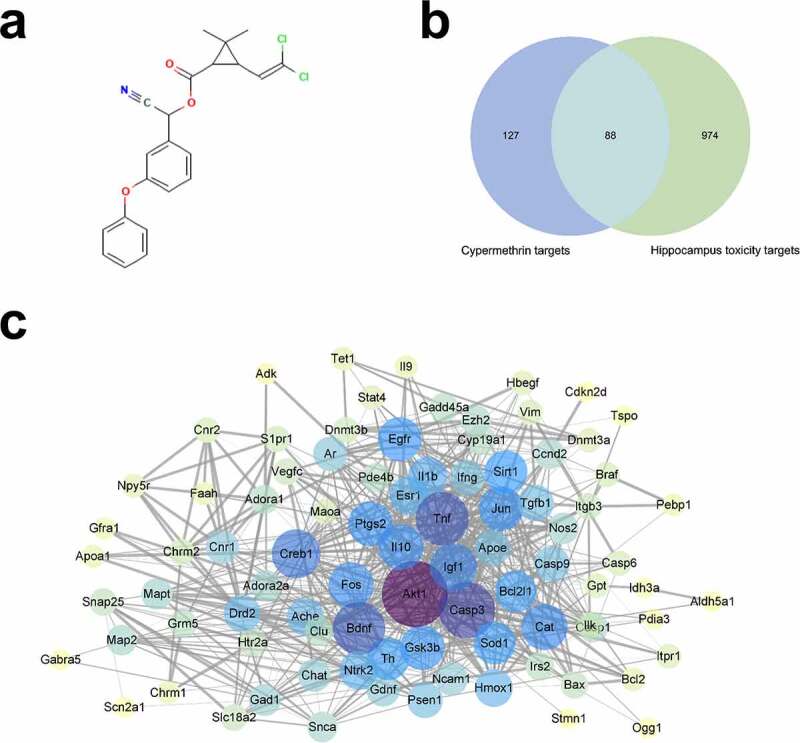
Candidate targets of CMN-induced hippocampal neurotoxicity and PPI network construction. (a) The 3D structure of CMN. (b) A Venn map of targets related to CMN-induced hippocampal neurotoxicity. (c) The PPI network. The width of each edge is proportional to the combined score, while the color (from yellow to purple) and size of each node are proportional to the degree of interaction

By uploading the 3D structure of CMN (Figure 1(a)), a total of 215 potential targets of CMN were identified from the SwissTargetPrediction, CTD, and STITCH databases after elimination of duplicates. Meanwhile, 1,062 targets related to hippocampal neurotoxicity were also obtained from the CTD and NCBI gene databases. Of the available data, 88 overlapping molecules were recognized as candidate targets that might participate in CMN-induced hippocampal neurotoxicity (Figure 1(b), Table S2). Additionally, the above targets were used to construct a PPI network with the String database. The PPI network included 88 nodes and 735 edges, with an average node degree of 16.7 (Figure 1(c), Table S3). Then, the network was imported into Cytoscape 3.6.0 software and analyzed with the Network Analyzer tool. A thicker edge indicated a higher combined score, while a deeper node color and larger size indicated a higher degree of interaction.

### GO and KEGG enrichment analyses

**Figure 2.**
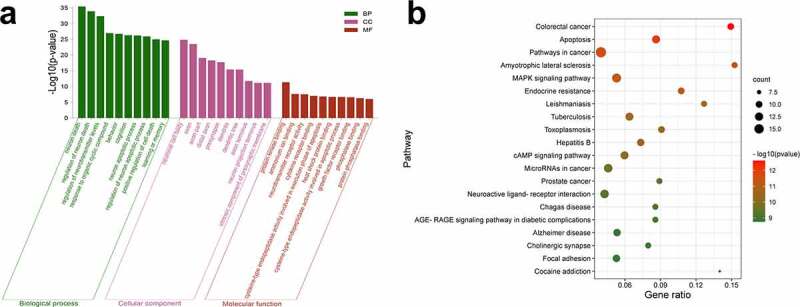
GO and KEGG enrichment analyses. (a) GO enrichment analysis. The Y-axis shows enriched *p*-values and the X-axis shows significantly enriched GO terms of the intersectional targets. (b) KEGG pathway enrichment analysis. The Y-axis shows significantly enriched KEGG pathways of the intersectional targets and the X-axis shows the gene ratio of these pathways

To investigate the potential mechanism underlying CMN-induced hippocampal neurotoxicity, GO and KEGG pathway enrichment analyses of 88 intersection targets were performed using the Metascape database. As shown in Figure 2(a) (Table S4), the BP domain was mainly enriched in the categories of neuron death, learning or memory, cognition, and neuron apoptotic process. The CC domain was mainly enriched in the categories of neuronal cell body, axon, presynapse, and neuron projection terminus. The MF domain was mainly enriched in the categories of protein kinase binding, neurotransmitter receptor activity, and cysteine-type endopeptidase activity involved in apoptotic process and phosphatase binding. The KEGG pathway analysis showed that the common targets were mainly enriched in apoptosis, amyotrophic lateral sclerosis (ALS), the MAPK signaling pathway, and Alzheimer’s disease (AD) (Figure 2(b), Table S5).

### Prediction of hub targets and construction of a CMN-targets-hippocampal neurotoxicity network

**Figure 3.**
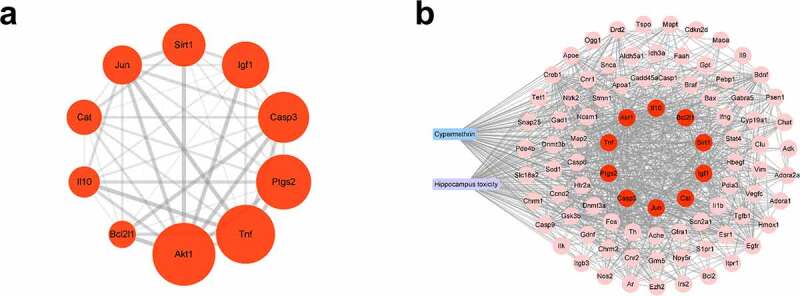
Prediction of hub targets and construction of a CMN-targets-hippocampal neurotoxicity network. (a) The MCC algorithm was used to identify the 10 hub targets. The width of each edge is proportional to the combined score and the size of each node is proportional to the degree of interaction. (b) The CMN-targets-hippocampal neurotoxicity network. The blue rectangle represents CMN, the purple rectangle represents hippocampal neurotoxicity, the red circles represent hub targets, and the pink circles represent other targets

To further explore the underlying mechanism of CMN-induced hippocampal neurotoxicity, the top 10 hub targets of the PPI network were determined using the MCC algorithm of the cytoHubba plugin of Cytoscape 3.6.0 software. In general, the hub targets ranked from highest to lowest were AKT serine/threonine kinase 1 (Akt1), tumor necrosis factor (Tnf), prostaglandin-endoperoxide synthase 2 (Ptgs2), caspase 3 (Casp3), insulin like growth factor 1 (Igf1), sirtuin 1 (Sirt1), Jun proto-oncogene (Jun), catalase (Cat), interleukin 10 (Il10) and Bcl2 like 1 (Bcl2l1) (Figure 3(a), Table S6). A chemical-target-specific phenotype network diagram was constructed using Cytoscape 3.6.0 software to present the relationships of CMN, targets, and hippocampal neurotoxicity. As shown in Figure 3(b), the network consisted of 90 nodes and 911 edges.

### Effects of CMN on cytotoxicity of HT22 cells

**Figure 4.**
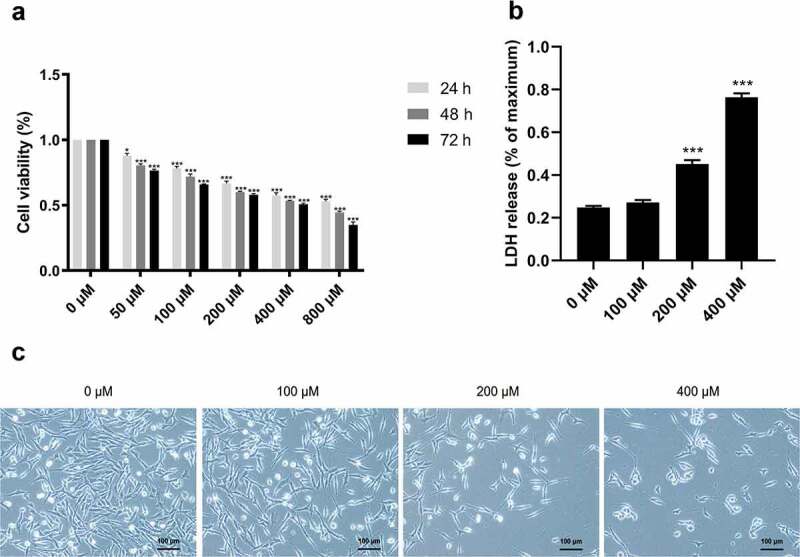
Effects of CMN on cytotoxicity of HT22 cells. (a) Cell viability was detected using the CCK-8 assay (b) LDH release was measured with an LDH assay kit. (c) Light microscopic images of morphological changes. Scar bar, 100 μm. Each column represents the mean ± SEM, n = 6. *p < 0.05 vs. 0 μM, **p < 0.01 vs. 0 μM, ***p < 0.001 vs. 0 μM

To investigate the neurotoxic effect of CMN in the hippocampus, HT22 cells were treated with various concentrations of CMN for 24, 48, and 72 h, and then subjected to the CCK-8 assay to assess cell viability. The results showed that CMN decreased the viability of HT22 cells in a time- and dose-dependent manner [Figure 4(a), ANOVA: group effect: F(5,72) = 3778, *p* < 0.001; time effect: F(2,72) = 418.8, *p* < 0.001; interaction effect: F(10,72) = 28.83, *p* < 0.001]. The half-maximal inhibitory concentrations of CMN in HT22 cells at 24, 48, and 72 h were 784.4, 502.9, and 340.7 μM, respectively. As the effects of pesticide exposure at low concentrations tended to materialize after prolonged periods, CMN concentrations of 0, 100, 200, and 400 μM for 72 h were selected in the following experiments. As compared with the group treated with 0 μM CMN, LDH release was significantly increased in the groups treated with 200 and 400 μM (Figure 4(b), ANOVA: F(3,16) = 1812, *p* < 0.001; 0 vs. 200 μM: *p* < 0.001, 0 vs. 400 μM: *p* < 0.001). The reduction in cell number and size increased with the CMN concentration (Figure 4(c)).

### Effects of CMN on apoptosis of HT22 cells

**Figure 5.**
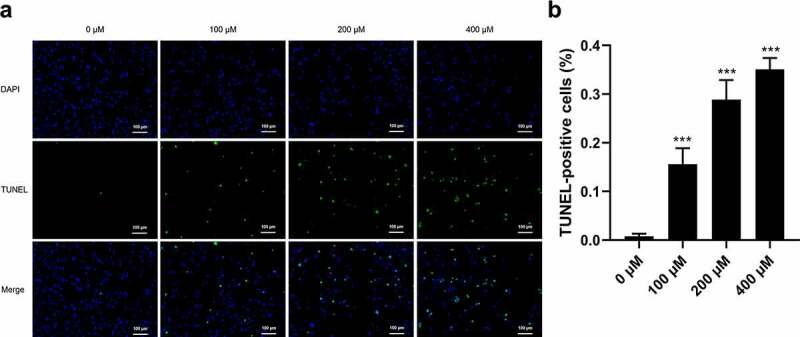
Effects of CMN on apoptosis of HT22 cells. (a) Apoptosis was detected using the TUNEL assay. Blue, 4ʹ,6-diamidino-2-phenylindole; green, TUNEL-positive cells. Scale bar, 100 µm. (b) The ratio of TUNEL-positive cells. Each column represents the mean ± SEM, n = 6. *p < 0.05 vs. 0 μM, **p < 0.01 vs. 0 μM, ***p < 0.001 vs. 0 μM

TUNEL staining was performed to determine whether CMN induces apoptosis of HT22 cells. As shown in Figure 5(a), only a small proportion of cells underwent apoptosis in the control group (0 μM CMN). Comparatively, the proportions of TUNEL-positive cells were significantly increased in the groups treated with 100, 200, and 400 μM CMN (Figure 5(b), ANOVA: F(3,20) = 171.90; all, *p* < 0.001).

### Effects of CMN on mRNA expression levels of hub targets in HT22 cells

**Figure 6.**
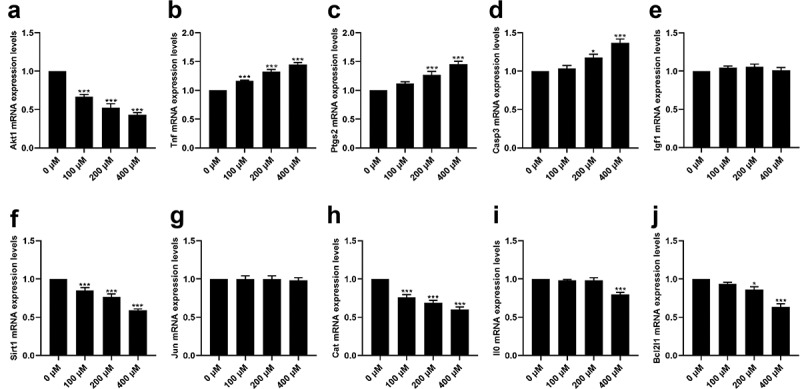
Effects of CMN on mRNA expression levels of hub targets in HT22 cells. (a) Akt1, (b) Tnf, (c) Ptgs2, (d) Casp3, (e) Igf1, (f) Sirt1, (g) Jun, (h) Cat, (i) Il10, and (j) Bcl2l1. Expression levels were standardized to β-actin. Each column represents the mean ± SEM, n = 6, *p < 0.05 vs. 0 μM, **p < 0.01 vs. 0 μM, ***p < 0.001 vs. 0 μM

As shown in Figure 6, CMN treatment significantly decreased the mRNA expression levels of Akt1 [ANOVA: F(3,20) = 55.10, *p* < 0.001; 0 vs. 100 μM: *p* < 0.001, 0 vs. 200 μM: *p* < 0.001, 0 vs. 400 μM: *p* < 0.001], Sirt1 (ANOVA: F(3,20) = 36.28, *p* < 0.001; 0 vs. 100 μM: *p* < 0.001, 0 vs. 200 μM: *p* < 0.001, 0 vs. 400 μM: *p* < 0.001), and Cat (ANOVA: F(3,20) = 32.03, *p* < 0.001; 0 vs. 100 μM: *p* < 0.001, 0 vs. 200 μM: *p* < 0.001, 0 vs. 400 μM: *p* < 0.001) in a dose-dependent manner (Figure 6(a,f,h)). The mRNA expression levels of Bcl2l1 were decreased in the groups treated with 200 and 400 μM CMN [Figure 6(j), ANOVA: F(3,20) = 29.03, *p* < 0.001; 0 vs. 200 μM: *p* < 0.05, 0 vs. 400 μM: *p* < 0.001], while mRNA expression levels of Il10 were only decreased in the group treated with 400 μM CMN [Figure 6(i), ANOVA: F(3,20) = 16.25, *p* < 0.001; 0 vs. 400 μM: *p* < 0.001]. There were no significant differences in the mRNA expression levels of Igf1 [ANOVA: F(3,20) = 1.22, *p* = 0.33] and Jun [ANOVA: F(3,20) = 0.05, *p* = 0.98] in any of the treatment groups as compared to the control groups (Figure 6(e,g)). The mRNA expression levels of Tnf increased in a dose-dependent manner [Figure 6(b), ANOVA: F(3,20) = 53.16, *p* < 0.001; 0 vs. 100 μM: *p* < 0.001, 0 vs. 200 μM: *p* < 0.001, 0 vs. 400 μM: *p* < 0.001], while the mRNA expression levels of Ptgs2 [ANOVA: F(3,20) = 20.51, *p* < 0.001; 0 μM vs. 200 μM: *p* < 0.001, 0 μM vs. 400 μM: *p* < 0.001) and Casp3 (ANOVA: F(3,20) = 19.92, *p* < 0.001; 0 μM vs. 200 μM: *p* < 0.05, 0 μM vs. 400 μM: *p* < 0.001] were increased in the groups treated with 200 and 400 μM (Figure 6(c,d)).

## Discussion

The pesticide CMN has been reported to cause neuronal damage in the hippocampus, and the underlying mechanism remains unclear [7]. In the current study, network pharmacology analysis was used to explore the mechanisms underlying the neurotoxic effect of CMN in the hippocampus, and the results were experimentally verified *in vitro* with using HT22 cells. In total, network pharmacology analysis identified 88 targets potentially associated with CMN-induced hippocampal neurotoxicity. Besides, the PPI network suggested that relationships among targets were intricate.

GO and KEGG enrichment analyses were performed to clarify the potential biological functions of the identified potential targets of CMN-induced hippocampal neurotoxicity. The results suggested that the BP categories of neuron apoptotic process, learning, and memory were significantly enriched. Notably, pesticide residues are strongly associated with neuronal apoptosis, and the hippocampus is critical for learning and memory [24]. With the CC categories, several neuronal structures were significantly enriched, and recent studies have established that CMN exposure could alter the morphology of primary hippocampal neurons [6]. Moreover, the MF categories of protein kinase binding, cytokine receptor binding, and phosphatase binding were also significantly enriched. Synaptic plasticity is closely related to hippocampal functions, which include ligand-receptor binding, kinase phosphorylation and activation [25]. Neurotoxicants can disrupt various biological processes, neural functions, and lead to neural injury [26].

Moreover, KEGG analysis revealed that the apoptosis and MAPK signaling pathways, which are strongly associated with hippocampal injury and AD, were significantly enriched. It is worth noting that several signaling pathways that might be irrelevant to neurotoxicity, such as those associated with leishmaniasis and focal adhesion, were also enriched. However, further studies are needed to confirm the role of these signaling pathways in CMN-induced hippocampal damage. Moreover, the top 10 hub targets identified by the MCC algorithm included Akt1, Tnf, Ptgs2, Casp3, Igf1, Sirt1, Jun, Cat, Il10, and Bcl2l1 [27]. Meanwhile, a network of CMN-targets-hippocampal neurotoxicity was constructed to further elucidate the potential mechanisms underlying the toxicological effects of CMN on the hippocampus.

*In vitro* experiments were conducted to further confirm the findings of network pharmacology analysis. The results of the CCK8 assay showed that CMN significantly inhibited the proliferation of HT22 cells in a time- and dose-dependent manner, which is consistent with the findings of a previous study [6]. Moreover, the results of the LDH assay showed that CMN significantly increased the release of LDH, which is an important parameter to evaluate cellular membrane integrity. In addition, CMN has been reported to induce damage to the membranes of various cell lines, such as HepG2 and SH-SY5Y [28,29]. The present study also showed that CMN induced apoptosis of HT22 cells in a dose-dependent manner, which is consistent with the enrichment in apoptosis, as determined by KEGG analysis.

In addition to Igf1 and Jun, the expression levels of eight other hub targets were altered to various degrees following exposure to CMN. For example, Akt1, Bcl2l1, Casp3, and Tnf were significantly enriched in apoptosis signaling pathways. CMN-induced apoptosis of primary hippocampal neurons contributes to hippocampal injury [6]. Moreover, environmental contaminants are known to alter the expression profiles of Bcl2l1, Cat, Casp3, and Tnf, which are involved in signaling pathways associated with ALS, which is characterized by synaptic dysfunction in the hippocampus [30,31]. Thus, we hypothesized that the neurotoxic effect of CMN could alter the signaling pathways associated with ALS. Remarkably, Casp3, Ptgs2, and Tnf have been identified as critical molecules in signaling pathways associated with AD. A recent study reported that CMN promoted the expression of markers in the hippocampus associated with the early onset of AD, suggesting that CMN exposure was a potential risk factor for AD [32].

Besides, the results showed that the mRNA expression levels of Sirt1 and Il10 were both decreased after CMN exposure. Sirt1, a member of the sirtuin family, is an important regulator of various biological processes [33], while IL10 is a critical anti-inflammatory cytokine produced by multiple cell types [34]. A previous study confirmed that CMN decreased the mRNA expression levels of Sirt1 in the germ cells of male rats [35]. However, CMN was also found to increase the mRNA expression levels of IL10 in rat striatum, which is contrary to results of the present study [36]. Considering the key roles of Sirt1 and Il0 in the hippocampus, CMN could act on these two targets and cause damage to the hippocampal neurons. Meanwhile, the detection results of Il10 suggested that neurotoxicity induced by CMN might be associated with inhibition of anti-inflammatory effects.

## Conclusion

The results of the current study demonstrated the neurotoxic effect of CMN on the hippocampus and clarified multiple signaling pathways engaged in CMN-induced hippocampal neurotoxicity, including those associated with apoptosis, ALS, and AD. Collectively, these findings provided a possible systemic strategy to investigate the mechanisms underlying CMN-induced hippocampal neurotoxicity. However, as a potential limitation to this study, the mechanisms *in vivo* remain to be further verified.

## Supplementary Material

Supplemental Material

## Data Availability

All data in this study are available from the corresponding authors upon request.
