# Supplementary material for: The effect and mechanism of cypermethrin-induced hippocampal neurotoxicity as determined by network pharmacology analysis and experimental validation
Source: Bioengineered. 2021 Nov 22;12(2):9279–89. doi: 10.1080/21655979.2021.2000106 (PMC8810029; doi:10.1080/21655979.2021.2000106)
Supplement: Supplemental Material [file KBIE_A_2000106_SM6064.doc]

**Table S1. Primers for RT-qPCR**

| **Gene symbol** | **Gene ID** | **Forward primer** | **Reverse primer** |
| --- | --- | --- | --- |
| β-actin | 11461 | GTGACGTTGACATCCGTAAAGA | GCCGGACTCATCGTACTCC |
| Akt1 | 11651 | ATGAACGACGTAGCCATTGTG | TTGTAGCCAATAAAGGTGCCAT |
| Tnf | 21926 | CTGAACTTCGGGGTGATCGG | GGCTTGTCACTCGAATTTTGAGA |
| Ptgs2 | 19225 | TGCACTATGGTTACAAAAGCTGG | TCAGGAAGCTCCTTATTTCCCTT |
| Casp3 | 12367 | CTGACTGGAAAGCCGAAACTC | CGACCCGTCCTTTGAATTTCT |
| Igf1 | 16000 | CACATCATGTCGTCTTCACACC | GGAAGCAACACTCATCCACAATG |
| Sirt1 | 93759 | GCTGACGACTTCGACGACG | TCGGTCAACAGGAGGTTGTCT |
| Jun | 16476 | TTCCTCCAGTCCGAGAGCG | TGAGAAGGTCCGAGTTCTTGG |
| Cat | 12359 | TGGCACACTTTGACAGAGAGC | CCTTTGCCTTGGAGTATCTGG |
| Il10 | 16153 | CTTACTGACTGGCATGAGGATCA | GCAGCTCTAGGAGCATGTGG |
| Bcl2l1 | 12048 | GACAAGGAGATGCAGGTATTGG | TCCCGTAGAGATCCACAAAAGT |

**Table S2. Information of targets related to hippocampal neurotoxicity induced by CMN**

| **Gene name** | **Uniprot ID** | **Protein names** |
| --- | --- | --- |
| Ache | P21836 | Acetylcholinesterase |
| Adk | P55264 | Adenosine kinase |
| Adora1 | Q60612 | Adenosine receptor A1 |
| Adora2a | Q60613 | Adenosine receptor A2a |
| Akt1 | P31750 | RAC-alpha serine/threonine-protein kinase |
| Aldh5a1 | Q8BWF0 | Succinate-semialdehyde dehydrogenase |
| Apoa1 | Q00623 | Apolipoprotein A-I |
| Apoe | P08226 | Apolipoprotein E |
| Ar | P19091 | Androgen receptor |
| Bax | Q07813 | Apoptosis regulator BAX |
| Bcl2 | P10417 | Apoptosis regulator Bcl-2 |
| Bcl2l1 | Q64373 | Bcl-2-like protein 1 |
| Bdnf | P21237 | Brain-derived neurotrophic factor |
| Braf | P28028 | Serine/threonine-protein kinase B-raf |
| Casp1 | P29452 | Caspase-1 |
| Casp3 | P70677 | Caspase-3 |
| Casp6 | O08738 | Caspase-6 |
| Casp9 | Q8C3Q9 | Caspase-9 |
| Cat | P24270 | Catalase |
| Ccnd2 | P30280 | G1/S-specific cyclin-D2 |
| Chat | Q03059 | Choline O-acetyltransferase |
| Chrm1 | P12657 | Muscarinic acetylcholine receptor M1 |
| Chrm2 | Q9ERZ4 | Muscarinic acetylcholine receptor M2 |
| Clu | Q06890 | Clusterin |
| Cnr1 | O88689 | Protocadherin alpha-4 |
| Cnr2 | P47936 | Cannabinoid receptor 2 |
| Creb1 | Q01147 | Cyclic AMP-responsive element-binding protein 1 |
| Cyp19a1 | P28649 | Aromatase |
| Dnmt3a | O88508 | DNA (cytosine-5)-methyltransferase 3A |
| Dnmt3b | O88509 | DNA (cytosine-5)-methyltransferase 3B |
| Drd2 | P61168 | D(2) dopamine receptor |
| Egfr | Q01279 | Epidermal growth factor receptor |
| Esr1 | P19785 | Estrogen receptor |
| Ezh2 | Q61188 | Histone-lysine N-methyltransferase EZH2 |
| Faah | O08914 | Fatty-acid amide hydrolase 1 |
| Fos | P01101 | Proto-oncogene c-Fos |
| Gabra5 | Q8BHJ7 | Gamma-aminobutyric acid receptor subunit alpha-5 |
| Gad1 | P48318 | Glutamate decarboxylase 1 |
| Gadd45a | P48316 | Growth arrest and DNA damage-inducible protein GADD45 alpha |
| Gdnf | P48540 | Glial cell line-derived neurotrophic factor |
| Gfra1 | P97785 | GDNF family receptor alpha-1 |
| Gpt | Q8QZR5 | Alanine aminotransferase 1 |
| Grm5 | Q3UVX5 | Metabotropic glutamate receptor 5 |
| Gsk3b | Q9WV60 | Glycogen synthase kinase-3 beta |
| Hbegf | Q06186 | Proheparin-binding EGF-like growth factor |
| Hmox1 | P14901 | Heme oxygenase 1 |
| Htr2a | P35363 | 5-hydroxytryptamine receptor 2A |
| Ifng | P01580 | Interferon gamma |
| Igf1 | P05017 | Insulin-like growth factor I |
| Il10 | P18893 | Interleukin-10 |
| Il1b | P10749 | Interleukin-1 beta |
| Il9 | P15247 | Interleukin-9 |
| Ilk | O55222 | Integrin-linked protein kinase |
| Irs2 | P81122 | Insulin receptor substrate 2 |
| Itgb3 | O54890 | Integrin beta-3 |
| Itpr1 | P11881 | Inositol 1,4,5-trisphosphate receptor type 1 |
| Jun | P05627 | Transcription factor AP-1 |
| Maoa | Q64133 | Amine oxidase |
| Map2 | P20357 | Microtubule-associated protein 2 |
| Mapt | P10637 | Microtubule-associated protein tau |
| Ncam1 | P13595 | Neural cell adhesion molecule 1 |
| Nos2 | P29477 | Nitric oxide synthase, inducible |
| Npy5r | O70342 | Neuropeptide Y receptor type 5 |
| Ntrk2 | P15209 | BDNF/NT-3 growth factors receptor |
| Ogg1 | O08760 | N-glycosylase/DNA lyase |
| Pde4b | B1AWC8 | Phosphodiesterase |
| Pdia3 | P27773 | Protein disulfide-isomerase A3 |
| Pebp1 | P70296 | Phosphatidylethanolamine-binding protein 1 |
| Psen1 | P49769 | Presenilin-1 |
| Ptgs2 | Q05769 | Prostaglandin G/H synthase 2 |
| S1pr1 | O08530 | Sphingosine 1-phosphate receptor 1 |
| Scn2a1 | B1AWN6 | Sodium channel protein type 2 subunit alpha |
| Sirt1 | Q923E4 | NAD-dependent protein deacetylase sirtuin-1 |
| Slc18a2 | Q8BRU6 | Synaptic vesicular amine transporter |
| Snap25 | P60879 | Synaptosomal-associated protein 25 |
| Snca | O55042 | Alpha-synuclein |
| Sod1 | P08228 | Superoxide dismutase [Cu-Zn] |
| Tgfb1 | P04202 | Transforming growth factor beta-1 proprotein |
| Th | P24529 | Tyrosine 3-monooxygenase |
| Tnf | P06804 | Tumor necrosis factor |
| Stmn1 | P54227 | Stathmin |
| Stat4 | P42228 | Signal transducer and activator of transcription 4 |
| Vim | P20152 | Vimentin |
| Vegfc | P97953 | Vascular endothelial growth factor C |
| Idh3a | Q9D6R2 | Isocitrate dehydrogenase [NAD] subunit alpha |
| Tet1 | Q3URK3 | Methylcytosine dioxygenase TET1 |
| Tspo | P50637 | Translocator protein |
| Cdkn2d | Q60773 | Cyclin-dependent kinase 4 inhibitor D |

**Table S3. Information of PPI network**

| **#node1** | **node2** | **node1_string_id** | **node2_string_id** | **neighborhood_on_chromosome** | **gene_fusion** | **phylogenetic_cooccurrence** | **homology** | **coexpression** | **experimentally_determined_interaction** | **database_annotated** | **automated_textmining** | **combined_score** |
| --- | --- | --- | --- | --- | --- | --- | --- | --- | --- | --- | --- | --- |
| Ache | Akt1 | 10090.ENSMUSP00000024099 | 10090.ENSMUSP00000001780 | 0 | 0 | 0 | 0 | 0 | 0.054 | 0 | 0.474 | 0.481 |
| Ache | Tnf | 10090.ENSMUSP00000024099 | 10090.ENSMUSP00000025263 | 0 | 0 | 0 | 0 | 0 | 0 | 0 | 0.449 | 0.449 |
| Ache | Ptgs2 | 10090.ENSMUSP00000024099 | 10090.ENSMUSP00000035065 | 0 | 0 | 0 | 0 | 0 | 0.056 | 0 | 0.446 | 0.454 |
| Ache | Grin2b | 10090.ENSMUSP00000024099 | 10090.ENSMUSP00000062284 | 0 | 0 | 0 | 0 | 0.102 | 0 | 0 | 0.424 | 0.461 |
| Ache | Gpx4 | 10090.ENSMUSP00000024099 | 10090.ENSMUSP00000094863 | 0 | 0 | 0 | 0 | 0 | 0 | 0 | 0.478 | 0.478 |
| Ache | Faah | 10090.ENSMUSP00000024099 | 10090.ENSMUSP00000041543 | 0 | 0 | 0 | 0 | 0.061 | 0 | 0 | 0.472 | 0.483 |
| Ache | Creb1 | 10090.ENSMUSP00000024099 | 10090.ENSMUSP00000140112 | 0 | 0 | 0 | 0 | 0 | 0 | 0 | 0.536 | 0.536 |
| Ache | Il6 | 10090.ENSMUSP00000024099 | 10090.ENSMUSP00000026845 | 0 | 0 | 0 | 0 | 0 | 0 | 0 | 0.537 | 0.537 |
| Ache | Grin1 | 10090.ENSMUSP00000024099 | 10090.ENSMUSP00000028335 | 0 | 0 | 0 | 0 | 0.125 | 0 | 0 | 0.546 | 0.586 |
| Ache | Bdnf | 10090.ENSMUSP00000024099 | 10090.ENSMUSP00000057989 | 0 | 0 | 0 | 0 | 0.052 | 0 | 0 | 0.633 | 0.637 |
| Ache | Ngf | 10090.ENSMUSP00000024099 | 10090.ENSMUSP00000102538 | 0 | 0 | 0 | 0 | 0 | 0 | 0 | 0.649 | 0.649 |
| Akt1 | Il4 | 10090.ENSMUSP00000001780 | 10090.ENSMUSP00000000889 | 0 | 0 | 0 | 0 | 0.062 | 0 | 0 | 0.671 | 0.679 |
| Akt1 | Gpx4 | 10090.ENSMUSP00000001780 | 10090.ENSMUSP00000094863 | 0 | 0 | 0 | 0 | 0 | 0.049 | 0 | 0.398 | 0.403 |
| Akt1 | Mif | 10090.ENSMUSP00000001780 | 10090.ENSMUSP00000041149 | 0 | 0 | 0 | 0 | 0 | 0.048 | 0 | 0.488 | 0.491 |
| Akt1 | Gpx1 | 10090.ENSMUSP00000001780 | 10090.ENSMUSP00000081010 | 0 | 0 | 0 | 0 | 0 | 0.049 | 0 | 0.503 | 0.507 |
| Akt1 | Nqo1 | 10090.ENSMUSP00000001780 | 10090.ENSMUSP00000003947 | 0 | 0 | 0 | 0 | 0 | 0 | 0 | 0.571 | 0.571 |
| Akt1 | Bax | 10090.ENSMUSP00000001780 | 10090.ENSMUSP00000033093 | 0 | 0 | 0 | 0 | 0 | 0.048 | 0 | 0.58 | 0.583 |
| Akt1 | Ifng | 10090.ENSMUSP00000001780 | 10090.ENSMUSP00000063800 | 0 | 0 | 0 | 0 | 0 | 0 | 0 | 0.588 | 0.588 |
| Akt1 | Grin1 | 10090.ENSMUSP00000001780 | 10090.ENSMUSP00000028335 | 0 | 0 | 0 | 0 | 0 | 0.046 | 0 | 0.602 | 0.604 |
| Akt1 | Grin2b | 10090.ENSMUSP00000001780 | 10090.ENSMUSP00000062284 | 0 | 0 | 0 | 0 | 0 | 0.068 | 0 | 0.701 | 0.709 |
| Akt1 | Il2 | 10090.ENSMUSP00000001780 | 10090.ENSMUSP00000029275 | 0 | 0 | 0 | 0 | 0 | 0 | 0 | 0.73 | 0.73 |
| Akt1 | Tlr4 | 10090.ENSMUSP00000001780 | 10090.ENSMUSP00000045770 | 0 | 0 | 0 | 0 | 0.062 | 0.128 | 0 | 0.7 | 0.733 |
| Akt1 | Bdnf | 10090.ENSMUSP00000001780 | 10090.ENSMUSP00000057989 | 0 | 0 | 0 | 0 | 0 | 0.197 | 0 | 0.765 | 0.804 |
| Akt1 | Fgf2 | 10090.ENSMUSP00000001780 | 10090.ENSMUSP00000037694 | 0 | 0 | 0 | 0 | 0.062 | 0.057 | 0 | 0.8 | 0.808 |
| Akt1 | Il10 | 10090.ENSMUSP00000001780 | 10090.ENSMUSP00000016673 | 0 | 0 | 0 | 0 | 0 | 0 | 0 | 0.835 | 0.835 |
| Akt1 | Ptgs2 | 10090.ENSMUSP00000001780 | 10090.ENSMUSP00000035065 | 0 | 0 | 0 | 0 | 0.062 | 0.056 | 0 | 0.845 | 0.851 |
| Akt1 | Hmox1 | 10090.ENSMUSP00000001780 | 10090.ENSMUSP00000005548 | 0 | 0 | 0 | 0 | 0 | 0.117 | 0 | 0.839 | 0.852 |
| Akt1 | Hspa5 | 10090.ENSMUSP00000001780 | 10090.ENSMUSP00000097747 | 0 | 0 | 0 | 0 | 0 | 0.197 | 0 | 0.83 | 0.858 |
| Akt1 | Ngf | 10090.ENSMUSP00000001780 | 10090.ENSMUSP00000102538 | 0 | 0 | 0 | 0 | 0 | 0.197 | 0 | 0.836 | 0.864 |
| Akt1 | Il6 | 10090.ENSMUSP00000001780 | 10090.ENSMUSP00000026845 | 0 | 0 | 0 | 0 | 0.054 | 0 | 0 | 0.895 | 0.897 |
| Akt1 | Tnf | 10090.ENSMUSP00000001780 | 10090.ENSMUSP00000025263 | 0 | 0 | 0 | 0 | 0 | 0.074 | 0 | 0.895 | 0.898 |
| Akt1 | Mapk1 | 10090.ENSMUSP00000001780 | 10090.ENSMUSP00000065983 | 0 | 0 | 0.265 | 0.593 | 0.064 | 0.15 | 0.8 | 0.884 | 0.899 |
| Akt1 | Mapk3 | 10090.ENSMUSP00000001780 | 10090.ENSMUSP00000051619 | 0 | 0 | 0.268 | 0.589 | 0.084 | 0.15 | 0.8 | 0.933 | 0.905 |
| Akt1 | Ccnd1 | 10090.ENSMUSP00000001780 | 10090.ENSMUSP00000091495 | 0 | 0 | 0 | 0 | 0.078 | 0.062 | 0 | 0.928 | 0.932 |
| Akt1 | Nos2 | 10090.ENSMUSP00000001780 | 10090.ENSMUSP00000018610 | 0 | 0 | 0 | 0 | 0.062 | 0.2 | 0.8 | 0.606 | 0.932 |
| Akt1 | Cd86 | 10090.ENSMUSP00000001780 | 10090.ENSMUSP00000087047 | 0 | 0 | 0 | 0 | 0.061 | 0 | 0.9 | 0.528 | 0.951 |
| Akt1 | Creb1 | 10090.ENSMUSP00000001780 | 10090.ENSMUSP00000140112 | 0 | 0 | 0 | 0 | 0.062 | 0.197 | 0.9 | 0.912 | 0.992 |
| Bax | Hmox1 | 10090.ENSMUSP00000033093 | 10090.ENSMUSP00000005548 | 0 | 0 | 0 | 0 | 0.084 | 0 | 0 | 0.399 | 0.426 |
| Bax | Tnf | 10090.ENSMUSP00000033093 | 10090.ENSMUSP00000025263 | 0 | 0 | 0 | 0 | 0 | 0.157 | 0 | 0.418 | 0.488 |
| Bax | Ccnd1 | 10090.ENSMUSP00000033093 | 10090.ENSMUSP00000091495 | 0 | 0 | 0 | 0 | 0 | 0 | 0 | 0.446 | 0.446 |
| Bche | Il6 | 10090.ENSMUSP00000029367 | 10090.ENSMUSP00000026845 | 0 | 0 | 0 | 0 | 0 | 0 | 0 | 0.419 | 0.419 |
| Bche | Grin1 | 10090.ENSMUSP00000029367 | 10090.ENSMUSP00000028335 | 0 | 0 | 0 | 0 | 0.062 | 0 | 0 | 0.455 | 0.467 |
| Bche | Gpx4 | 10090.ENSMUSP00000029367 | 10090.ENSMUSP00000094863 | 0 | 0 | 0 | 0 | 0 | 0 | 0 | 0.442 | 0.442 |
| Bche | Bdnf | 10090.ENSMUSP00000029367 | 10090.ENSMUSP00000057989 | 0 | 0 | 0 | 0 | 0.052 | 0 | 0 | 0.48 | 0.486 |
| Bche | Ngf | 10090.ENSMUSP00000029367 | 10090.ENSMUSP00000102538 | 0 | 0 | 0 | 0 | 0 | 0 | 0 | 0.535 | 0.535 |
| Bdnf | Il4 | 10090.ENSMUSP00000057989 | 10090.ENSMUSP00000000889 | 0 | 0 | 0 | 0 | 0 | 0 | 0 | 0.571 | 0.571 |
| Bdnf | Hmox1 | 10090.ENSMUSP00000057989 | 10090.ENSMUSP00000005548 | 0 | 0 | 0 | 0 | 0 | 0 | 0 | 0.462 | 0.462 |
| Bdnf | Il10 | 10090.ENSMUSP00000057989 | 10090.ENSMUSP00000016673 | 0 | 0 | 0 | 0 | 0 | 0 | 0 | 0.612 | 0.612 |
| Bdnf | Nos2 | 10090.ENSMUSP00000057989 | 10090.ENSMUSP00000018610 | 0 | 0 | 0 | 0 | 0.061 | 0.141 | 0 | 0.374 | 0.451 |
| Bdnf | Slc6a3 | 10090.ENSMUSP00000057989 | 10090.ENSMUSP00000022100 | 0 | 0 | 0 | 0 | 0.06 | 0 | 0 | 0.504 | 0.514 |
| Bdnf | Tnf | 10090.ENSMUSP00000057989 | 10090.ENSMUSP00000025263 | 0 | 0 | 0 | 0 | 0 | 0 | 0 | 0.713 | 0.713 |
| Bdnf | Il6 | 10090.ENSMUSP00000057989 | 10090.ENSMUSP00000026845 | 0 | 0 | 0 | 0 | 0 | 0 | 0 | 0.804 | 0.805 |
| Bdnf | Grin1 | 10090.ENSMUSP00000057989 | 10090.ENSMUSP00000028335 | 0 | 0 | 0 | 0 | 0.082 | 0 | 0 | 0.731 | 0.742 |
| Bdnf | Ptgs2 | 10090.ENSMUSP00000057989 | 10090.ENSMUSP00000035065 | 0 | 0 | 0 | 0 | 0.069 | 0 | 0 | 0.525 | 0.539 |
| Bdnf | Fgf2 | 10090.ENSMUSP00000057989 | 10090.ENSMUSP00000037694 | 0 | 0 | 0 | 0 | 0.062 | 0 | 0 | 0.904 | 0.907 |
| Bdnf | Tlr4 | 10090.ENSMUSP00000057989 | 10090.ENSMUSP00000045770 | 0 | 0 | 0 | 0 | 0 | 0.096 | 0 | 0.433 | 0.466 |
| Bdnf | Camk4 | 10090.ENSMUSP00000057989 | 10090.ENSMUSP00000046539 | 0 | 0 | 0 | 0 | 0.062 | 0 | 0 | 0.544 | 0.554 |
| Bdnf | Mapk3 | 10090.ENSMUSP00000057989 | 10090.ENSMUSP00000051619 | 0 | 0 | 0 | 0 | 0 | 0 | 0 | 0.66 | 0.66 |
| Bdnf | Nefh | 10090.ENSMUSP00000057989 | 10090.ENSMUSP00000091061 | 0 | 0 | 0 | 0 | 0.082 | 0 | 0 | 0.522 | 0.542 |
| Bdnf | Mapk1 | 10090.ENSMUSP00000057989 | 10090.ENSMUSP00000065983 | 0 | 0 | 0 | 0 | 0 | 0 | 0 | 0.702 | 0.702 |
| Bdnf | Grin2b | 10090.ENSMUSP00000057989 | 10090.ENSMUSP00000062284 | 0 | 0 | 0 | 0 | 0.081 | 0 | 0 | 0.765 | 0.775 |
| Bdnf | Creb1 | 10090.ENSMUSP00000057989 | 10090.ENSMUSP00000140112 | 0 | 0 | 0 | 0 | 0 | 0 | 0 | 0.942 | 0.942 |
| Camk4 | Il4 | 10090.ENSMUSP00000046539 | 10090.ENSMUSP00000000889 | 0 | 0 | 0 | 0 | 0.062 | 0 | 0 | 0.451 | 0.463 |
| Camk4 | Hmox1 | 10090.ENSMUSP00000046539 | 10090.ENSMUSP00000005548 | 0 | 0 | 0 | 0 | 0.083 | 0 | 0 | 0.448 | 0.473 |
| Camk4 | Grin1 | 10090.ENSMUSP00000046539 | 10090.ENSMUSP00000028335 | 0 | 0 | 0 | 0 | 0.168 | 0 | 0 | 0.609 | 0.661 |
| Camk4 | Grin2b | 10090.ENSMUSP00000046539 | 10090.ENSMUSP00000062284 | 0 | 0 | 0 | 0 | 0.176 | 0 | 0 | 0.488 | 0.56 |
| Camk4 | Creb1 | 10090.ENSMUSP00000046539 | 10090.ENSMUSP00000140112 | 0 | 0 | 0 | 0 | 0 | 0.154 | 0.9 | 0.886 | 0.989 |
| Ccnd1 | Il4 | 10090.ENSMUSP00000091495 | 10090.ENSMUSP00000000889 | 0 | 0 | 0 | 0 | 0 | 0 | 0 | 0.46 | 0.46 |
| Ccnd1 | Nqo1 | 10090.ENSMUSP00000091495 | 10090.ENSMUSP00000003947 | 0 | 0 | 0 | 0 | 0.082 | 0 | 0 | 0.395 | 0.421 |
| Ccnd1 | Hmox1 | 10090.ENSMUSP00000091495 | 10090.ENSMUSP00000005548 | 0 | 0 | 0 | 0 | 0.071 | 0 | 0 | 0.443 | 0.46 |
| Ccnd1 | Il10 | 10090.ENSMUSP00000091495 | 10090.ENSMUSP00000016673 | 0 | 0 | 0 | 0 | 0 | 0 | 0 | 0.436 | 0.436 |
| Ccnd1 | Tnf | 10090.ENSMUSP00000091495 | 10090.ENSMUSP00000025263 | 0 | 0 | 0 | 0 | 0 | 0 | 0 | 0.658 | 0.658 |
| Ccnd1 | Il6 | 10090.ENSMUSP00000091495 | 10090.ENSMUSP00000026845 | 0 | 0 | 0 | 0 | 0 | 0 | 0 | 0.811 | 0.811 |
| Ccnd1 | Il2 | 10090.ENSMUSP00000091495 | 10090.ENSMUSP00000029275 | 0 | 0 | 0 | 0 | 0 | 0 | 0 | 0.447 | 0.447 |
| Ccnd1 | Ptgs2 | 10090.ENSMUSP00000091495 | 10090.ENSMUSP00000035065 | 0 | 0 | 0 | 0 | 0.054 | 0 | 0 | 0.686 | 0.69 |
| Ccnd1 | Fgf2 | 10090.ENSMUSP00000091495 | 10090.ENSMUSP00000037694 | 0 | 0 | 0 | 0 | 0.061 | 0 | 0 | 0.795 | 0.799 |
| Ccnd1 | Mapk3 | 10090.ENSMUSP00000091495 | 10090.ENSMUSP00000051619 | 0 | 0 | 0 | 0 | 0 | 0.153 | 0 | 0.785 | 0.81 |
| Ccnd1 | Mapk1 | 10090.ENSMUSP00000091495 | 10090.ENSMUSP00000065983 | 0 | 0 | 0 | 0 | 0 | 0.167 | 0 | 0.774 | 0.804 |
| Ccnd1 | Ngf | 10090.ENSMUSP00000091495 | 10090.ENSMUSP00000102538 | 0 | 0 | 0 | 0 | 0.074 | 0 | 0 | 0.409 | 0.43 |
| Ccnd1 | Hspa5 | 10090.ENSMUSP00000091495 | 10090.ENSMUSP00000097747 | 0 | 0 | 0 | 0 | 0 | 0.047 | 0 | 0.448 | 0.451 |
| Ccnd1 | Creb1 | 10090.ENSMUSP00000091495 | 10090.ENSMUSP00000140112 | 0 | 0 | 0 | 0 | 0 | 0 | 0 | 0.608 | 0.608 |
| Cd86 | Il4 | 10090.ENSMUSP00000087047 | 10090.ENSMUSP00000000889 | 0 | 0 | 0 | 0 | 0.062 | 0 | 0 | 0.852 | 0.855 |
| Cd86 | Il10 | 10090.ENSMUSP00000087047 | 10090.ENSMUSP00000016673 | 0 | 0 | 0 | 0 | 0.062 | 0 | 0 | 0.919 | 0.921 |
| Cd86 | Nos2 | 10090.ENSMUSP00000087047 | 10090.ENSMUSP00000018610 | 0 | 0 | 0 | 0 | 0.061 | 0 | 0 | 0.721 | 0.727 |
| Cd86 | Tnf | 10090.ENSMUSP00000087047 | 10090.ENSMUSP00000025263 | 0 | 0 | 0 | 0 | 0.133 | 0.048 | 0 | 0.834 | 0.851 |
| Cd86 | Il6 | 10090.ENSMUSP00000087047 | 10090.ENSMUSP00000026845 | 0 | 0 | 0 | 0 | 0.101 | 0 | 0 | 0.837 | 0.847 |
| Cd86 | Il2 | 10090.ENSMUSP00000087047 | 10090.ENSMUSP00000029275 | 0 | 0 | 0 | 0 | 0 | 0 | 0 | 0.846 | 0.846 |
| Cd86 | Ptgs2 | 10090.ENSMUSP00000087047 | 10090.ENSMUSP00000035065 | 0 | 0 | 0 | 0 | 0.061 | 0 | 0 | 0.418 | 0.43 |
| Cd86 | Tlr4 | 10090.ENSMUSP00000087047 | 10090.ENSMUSP00000045770 | 0 | 0 | 0 | 0 | 0.125 | 0 | 0 | 0.859 | 0.872 |
| Cd86 | Mapk3 | 10090.ENSMUSP00000087047 | 10090.ENSMUSP00000051619 | 0 | 0 | 0 | 0 | 0 | 0 | 0 | 0.441 | 0.441 |
| Cd86 | Ifng | 10090.ENSMUSP00000087047 | 10090.ENSMUSP00000063800 | 0 | 0 | 0 | 0 | 0.171 | 0 | 0 | 0.868 | 0.886 |
| Cnr2 | Ptgs2 | 10090.ENSMUSP00000095454 | 10090.ENSMUSP00000035065 | 0 | 0 | 0 | 0 | 0 | 0.072 | 0 | 0.38 | 0.4 |
| Cnr2 | Faah | 10090.ENSMUSP00000095454 | 10090.ENSMUSP00000041543 | 0 | 0 | 0 | 0 | 0 | 0 | 0 | 0.907 | 0.907 |
| Creb1 | Il4 | 10090.ENSMUSP00000140112 | 10090.ENSMUSP00000000889 | 0 | 0 | 0 | 0 | 0 | 0 | 0 | 0.447 | 0.447 |
| Creb1 | Hmox1 | 10090.ENSMUSP00000140112 | 10090.ENSMUSP00000005548 | 0 | 0 | 0 | 0 | 0 | 0 | 0 | 0.548 | 0.548 |
| Creb1 | Il10 | 10090.ENSMUSP00000140112 | 10090.ENSMUSP00000016673 | 0 | 0 | 0 | 0 | 0 | 0 | 0 | 0.761 | 0.761 |
| Creb1 | Tnf | 10090.ENSMUSP00000140112 | 10090.ENSMUSP00000025263 | 0 | 0 | 0 | 0 | 0 | 0 | 0 | 0.644 | 0.644 |
| Creb1 | Il6 | 10090.ENSMUSP00000140112 | 10090.ENSMUSP00000026845 | 0 | 0 | 0 | 0 | 0 | 0 | 0 | 0.802 | 0.802 |
| Creb1 | Grin1 | 10090.ENSMUSP00000140112 | 10090.ENSMUSP00000028335 | 0 | 0 | 0 | 0 | 0 | 0 | 0 | 0.731 | 0.731 |
| Creb1 | Il2 | 10090.ENSMUSP00000140112 | 10090.ENSMUSP00000029275 | 0 | 0 | 0 | 0 | 0 | 0.121 | 0 | 0.587 | 0.621 |
| Creb1 | Ptgs2 | 10090.ENSMUSP00000140112 | 10090.ENSMUSP00000035065 | 0 | 0 | 0 | 0 | 0.051 | 0 | 0 | 0.76 | 0.763 |
| Creb1 | Fgf2 | 10090.ENSMUSP00000140112 | 10090.ENSMUSP00000037694 | 0 | 0 | 0 | 0 | 0 | 0 | 0 | 0.543 | 0.543 |
| Creb1 | Tlr4 | 10090.ENSMUSP00000140112 | 10090.ENSMUSP00000045770 | 0 | 0 | 0 | 0 | 0 | 0 | 0 | 0.527 | 0.527 |
| Creb1 | Mapk3 | 10090.ENSMUSP00000140112 | 10090.ENSMUSP00000051619 | 0 | 0 | 0 | 0 | 0 | 0.124 | 0.6 | 0.811 | 0.928 |
| Creb1 | Grin2b | 10090.ENSMUSP00000140112 | 10090.ENSMUSP00000062284 | 0 | 0 | 0 | 0 | 0 | 0 | 0 | 0.822 | 0.822 |
| Creb1 | Ifng | 10090.ENSMUSP00000140112 | 10090.ENSMUSP00000063800 | 0 | 0 | 0 | 0 | 0 | 0 | 0 | 0.605 | 0.605 |
| Creb1 | Mapk1 | 10090.ENSMUSP00000140112 | 10090.ENSMUSP00000065983 | 0 | 0 | 0 | 0 | 0 | 0.124 | 0.6 | 0.738 | 0.9 |
| Creb1 | Hspa5 | 10090.ENSMUSP00000140112 | 10090.ENSMUSP00000097747 | 0 | 0 | 0 | 0 | 0 | 0.155 | 0 | 0.395 | 0.466 |
| Creb1 | Ngf | 10090.ENSMUSP00000140112 | 10090.ENSMUSP00000102538 | 0 | 0 | 0 | 0 | 0 | 0 | 0 | 0.696 | 0.696 |
| Faah | Ptgs2 | 10090.ENSMUSP00000041543 | 10090.ENSMUSP00000035065 | 0 | 0 | 0 | 0 | 0 | 0 | 0 | 0.634 | 0.634 |
| Fgf2 | Il4 | 10090.ENSMUSP00000037694 | 10090.ENSMUSP00000000889 | 0 | 0 | 0 | 0 | 0.062 | 0 | 0 | 0.558 | 0.568 |
| Fgf2 | Hmox1 | 10090.ENSMUSP00000037694 | 10090.ENSMUSP00000005548 | 0 | 0 | 0 | 0 | 0 | 0 | 0 | 0.44 | 0.44 |
| Fgf2 | Il10 | 10090.ENSMUSP00000037694 | 10090.ENSMUSP00000016673 | 0 | 0 | 0 | 0 | 0 | 0 | 0 | 0.649 | 0.649 |
| Fgf2 | Tnf | 10090.ENSMUSP00000037694 | 10090.ENSMUSP00000025263 | 0 | 0 | 0 | 0 | 0 | 0 | 0 | 0.699 | 0.699 |
| Fgf2 | Il6 | 10090.ENSMUSP00000037694 | 10090.ENSMUSP00000026845 | 0 | 0 | 0 | 0 | 0.06 | 0 | 0 | 0.849 | 0.853 |
| Fgf2 | Il2 | 10090.ENSMUSP00000037694 | 10090.ENSMUSP00000029275 | 0 | 0 | 0 | 0 | 0 | 0 | 0 | 0.551 | 0.551 |
| Fgf2 | Ptgs2 | 10090.ENSMUSP00000037694 | 10090.ENSMUSP00000035065 | 0 | 0 | 0 | 0 | 0.056 | 0 | 0 | 0.733 | 0.737 |
| Fgf2 | Nefh | 10090.ENSMUSP00000037694 | 10090.ENSMUSP00000091061 | 0 | 0 | 0 | 0 | 0 | 0 | 0 | 0.425 | 0.424 |
| Fgf2 | Ifng | 10090.ENSMUSP00000037694 | 10090.ENSMUSP00000063800 | 0 | 0 | 0 | 0 | 0 | 0 | 0 | 0.592 | 0.592 |
| Fgf2 | Tlr4 | 10090.ENSMUSP00000037694 | 10090.ENSMUSP00000045770 | 0 | 0 | 0 | 0 | 0.062 | 0.05 | 0 | 0.621 | 0.633 |
| Fgf2 | Ngf | 10090.ENSMUSP00000037694 | 10090.ENSMUSP00000102538 | 0 | 0 | 0 | 0 | 0.088 | 0 | 0 | 0.877 | 0.883 |
| Fgf2 | Mapk1 | 10090.ENSMUSP00000037694 | 10090.ENSMUSP00000065983 | 0 | 0 | 0 | 0 | 0 | 0.072 | 0.9 | 0.72 | 0.971 |
| Fgf2 | Mapk3 | 10090.ENSMUSP00000037694 | 10090.ENSMUSP00000051619 | 0 | 0 | 0 | 0 | 0 | 0.072 | 0.9 | 0.723 | 0.972 |
| Gpx1 | Nqo1 | 10090.ENSMUSP00000081010 | 10090.ENSMUSP00000003947 | 0 | 0 | 0 | 0 | 0 | 0 | 0 | 0.687 | 0.687 |
| Gpx1 | Hmox1 | 10090.ENSMUSP00000081010 | 10090.ENSMUSP00000005548 | 0 | 0 | 0 | 0 | 0.066 | 0.161 | 0 | 0.744 | 0.782 |
| Gpx1 | Tnf | 10090.ENSMUSP00000081010 | 10090.ENSMUSP00000025263 | 0 | 0 | 0 | 0 | 0 | 0 | 0 | 0.446 | 0.446 |
| Gpx1 | Il6 | 10090.ENSMUSP00000081010 | 10090.ENSMUSP00000026845 | 0 | 0 | 0 | 0 | 0 | 0 | 0 | 0.536 | 0.536 |
| Gpx1 | Gsta2 | 10090.ENSMUSP00000081010 | 10090.ENSMUSP00000034902 | 0 | 0 | 0 | 0 | 0.061 | 0 | 0.65 | 0.319 | 0.756 |
| Gpx1 | Hspa5 | 10090.ENSMUSP00000081010 | 10090.ENSMUSP00000097747 | 0.041 | 0 | 0 | 0 | 0.117 | 0.072 | 0 | 0.344 | 0.415 |
| Gpx4 | Nqo1 | 10090.ENSMUSP00000094863 | 10090.ENSMUSP00000003947 | 0 | 0 | 0 | 0 | 0 | 0 | 0 | 0.616 | 0.616 |
| Gpx4 | Hmox1 | 10090.ENSMUSP00000094863 | 10090.ENSMUSP00000005548 | 0 | 0 | 0 | 0 | 0 | 0.161 | 0 | 0.518 | 0.578 |
| Gpx4 | Tnf | 10090.ENSMUSP00000094863 | 10090.ENSMUSP00000025263 | 0 | 0 | 0 | 0 | 0 | 0 | 0 | 0.404 | 0.404 |
| Gpx4 | Gsta2 | 10090.ENSMUSP00000094863 | 10090.ENSMUSP00000034902 | 0 | 0 | 0 | 0 | 0.061 | 0 | 0.65 | 0.293 | 0.747 |
| Gpx4 | Ptgs2 | 10090.ENSMUSP00000094863 | 10090.ENSMUSP00000035065 | 0 | 0 | 0 | 0 | 0 | 0 | 0 | 0.408 | 0.408 |
| Grin1 | Ngf | 10090.ENSMUSP00000028335 | 10090.ENSMUSP00000102538 | 0 | 0 | 0 | 0 | 0.062 | 0 | 0 | 0.482 | 0.493 |
| Grin1 | Tlr4 | 10090.ENSMUSP00000028335 | 10090.ENSMUSP00000045770 | 0 | 0 | 0 | 0 | 0 | 0.075 | 0 | 0.568 | 0.584 |
| Grin1 | Mapk1 | 10090.ENSMUSP00000028335 | 10090.ENSMUSP00000065983 | 0 | 0 | 0 | 0 | 0.06 | 0.392 | 0 | 0.466 | 0.668 |
| Grin1 | Mapk3 | 10090.ENSMUSP00000028335 | 10090.ENSMUSP00000051619 | 0 | 0 | 0 | 0 | 0.06 | 0.392 | 0 | 0.634 | 0.773 |
| Grin1 | Grin2b | 10090.ENSMUSP00000028335 | 10090.ENSMUSP00000062284 | 0 | 0 | 0 | 0.697 | 0.265 | 0.86 | 0.9 | 0.969 | 0.992 |
| Grin2b | Mapk3 | 10090.ENSMUSP00000062284 | 10090.ENSMUSP00000051619 | 0 | 0 | 0 | 0 | 0.053 | 0.357 | 0 | 0.539 | 0.694 |
| Grin2b | Ngf | 10090.ENSMUSP00000062284 | 10090.ENSMUSP00000102538 | 0 | 0 | 0 | 0 | 0 | 0 | 0 | 0.487 | 0.487 |
| Grin2b | Mapk1 | 10090.ENSMUSP00000062284 | 10090.ENSMUSP00000065983 | 0 | 0 | 0 | 0 | 0.053 | 0.357 | 0 | 0.353 | 0.572 |
| Gsta2 | Nqo1 | 10090.ENSMUSP00000034902 | 10090.ENSMUSP00000003947 | 0 | 0 | 0 | 0 | 0.079 | 0 | 0 | 0.596 | 0.612 |
| Gsta2 | Hmox1 | 10090.ENSMUSP00000034902 | 10090.ENSMUSP00000005548 | 0 | 0 | 0 | 0 | 0 | 0 | 0 | 0.458 | 0.457 |
| Hmox1 | Il4 | 10090.ENSMUSP00000005548 | 10090.ENSMUSP00000000889 | 0 | 0 | 0 | 0 | 0 | 0 | 0 | 0.522 | 0.522 |
| Hmox1 | Nqo1 | 10090.ENSMUSP00000005548 | 10090.ENSMUSP00000003947 | 0 | 0 | 0 | 0 | 0.088 | 0 | 0 | 0.919 | 0.924 |
| Hmox1 | Il2 | 10090.ENSMUSP00000005548 | 10090.ENSMUSP00000029275 | 0 | 0 | 0 | 0 | 0 | 0 | 0 | 0.445 | 0.445 |
| Hmox1 | Ifng | 10090.ENSMUSP00000005548 | 10090.ENSMUSP00000063800 | 0 | 0 | 0 | 0 | 0 | 0 | 0 | 0.53 | 0.53 |
| Hmox1 | Hspa5 | 10090.ENSMUSP00000005548 | 10090.ENSMUSP00000097747 | 0 | 0 | 0 | 0 | 0.066 | 0 | 0 | 0.531 | 0.544 |
| Hmox1 | Mapk3 | 10090.ENSMUSP00000005548 | 10090.ENSMUSP00000051619 | 0 | 0 | 0 | 0 | 0 | 0 | 0 | 0.641 | 0.641 |
| Hmox1 | Ngf | 10090.ENSMUSP00000005548 | 10090.ENSMUSP00000102538 | 0 | 0 | 0 | 0 | 0.062 | 0 | 0 | 0.638 | 0.646 |
| Hmox1 | Tnf | 10090.ENSMUSP00000005548 | 10090.ENSMUSP00000025263 | 0 | 0 | 0 | 0 | 0.067 | 0 | 0 | 0.73 | 0.737 |
| Hmox1 | Tlr4 | 10090.ENSMUSP00000005548 | 10090.ENSMUSP00000045770 | 0.042 | 0 | 0 | 0 | 0.056 | 0 | 0 | 0.794 | 0.797 |
| Hmox1 | Nos2 | 10090.ENSMUSP00000005548 | 10090.ENSMUSP00000018610 | 0 | 0 | 0 | 0 | 0 | 0.251 | 0 | 0.779 | 0.828 |
| Hmox1 | Ptgs2 | 10090.ENSMUSP00000005548 | 10090.ENSMUSP00000035065 | 0 | 0 | 0 | 0 | 0.085 | 0 | 0 | 0.84 | 0.848 |
| Hmox1 | Il6 | 10090.ENSMUSP00000005548 | 10090.ENSMUSP00000026845 | 0 | 0 | 0 | 0 | 0.061 | 0 | 0 | 0.845 | 0.848 |
| Hmox1 | Il10 | 10090.ENSMUSP00000005548 | 10090.ENSMUSP00000016673 | 0 | 0 | 0 | 0 | 0 | 0 | 0 | 0.878 | 0.878 |
| Hspa5 | Tnf | 10090.ENSMUSP00000097747 | 10090.ENSMUSP00000025263 | 0 | 0 | 0 | 0 | 0 | 0.094 | 0 | 0.574 | 0.597 |
| Hspa5 | Il6 | 10090.ENSMUSP00000097747 | 10090.ENSMUSP00000026845 | 0 | 0 | 0 | 0 | 0 | 0 | 0 | 0.52 | 0.52 |
| Hspa5 | Tlr4 | 10090.ENSMUSP00000097747 | 10090.ENSMUSP00000045770 | 0 | 0 | 0 | 0 | 0.045 | 0.061 | 0 | 0.598 | 0.608 |
| Hspa5 | Mapk3 | 10090.ENSMUSP00000097747 | 10090.ENSMUSP00000051619 | 0 | 0 | 0 | 0 | 0.061 | 0.207 | 0 | 0.565 | 0.648 |
| Hspa5 | Mapk1 | 10090.ENSMUSP00000097747 | 10090.ENSMUSP00000065983 | 0 | 0 | 0 | 0 | 0.061 | 0.197 | 0 | 0.306 | 0.431 |
| Ifng | Il4 | 10090.ENSMUSP00000063800 | 10090.ENSMUSP00000000889 | 0 | 0 | 0 | 0 | 0 | 0 | 0 | 0.921 | 0.921 |
| Ifng | Il10 | 10090.ENSMUSP00000063800 | 10090.ENSMUSP00000016673 | 0 | 0 | 0 | 0 | 0.082 | 0 | 0 | 0.954 | 0.956 |
| Ifng | Nos2 | 10090.ENSMUSP00000063800 | 10090.ENSMUSP00000018610 | 0 | 0 | 0 | 0 | 0 | 0 | 0 | 0.904 | 0.904 |
| Ifng | Tnf | 10090.ENSMUSP00000063800 | 10090.ENSMUSP00000025263 | 0 | 0 | 0 | 0 | 0.062 | 0.197 | 0 | 0.952 | 0.961 |
| Ifng | Il6 | 10090.ENSMUSP00000063800 | 10090.ENSMUSP00000026845 | 0 | 0 | 0 | 0 | 0.062 | 0 | 0 | 0.935 | 0.938 |
| Ifng | Il2 | 10090.ENSMUSP00000063800 | 10090.ENSMUSP00000029275 | 0 | 0 | 0 | 0 | 0.061 | 0 | 0 | 0.949 | 0.951 |
| Ifng | Ptgs2 | 10090.ENSMUSP00000063800 | 10090.ENSMUSP00000035065 | 0 | 0 | 0 | 0 | 0 | 0 | 0 | 0.642 | 0.642 |
| Ifng | Mif | 10090.ENSMUSP00000063800 | 10090.ENSMUSP00000041149 | 0 | 0 | 0 | 0 | 0 | 0 | 0 | 0.521 | 0.521 |
| Ifng | Tlr4 | 10090.ENSMUSP00000063800 | 10090.ENSMUSP00000045770 | 0 | 0 | 0 | 0 | 0 | 0 | 0 | 0.835 | 0.835 |
| Ifng | Mapk3 | 10090.ENSMUSP00000063800 | 10090.ENSMUSP00000051619 | 0 | 0 | 0 | 0 | 0 | 0 | 0 | 0.535 | 0.535 |
| Ifng | Ngf | 10090.ENSMUSP00000063800 | 10090.ENSMUSP00000102538 | 0 | 0 | 0 | 0 | 0 | 0 | 0 | 0.64 | 0.64 |
| Il10 | Il4 | 10090.ENSMUSP00000016673 | 10090.ENSMUSP00000000889 | 0 | 0 | 0 | 0 | 0 | 0 | 0 | 0.968 | 0.968 |
| Il10 | Ngf | 10090.ENSMUSP00000016673 | 10090.ENSMUSP00000102538 | 0 | 0 | 0 | 0 | 0 | 0 | 0 | 0.612 | 0.612 |
| Il10 | Mapk1 | 10090.ENSMUSP00000016673 | 10090.ENSMUSP00000065983 | 0 | 0 | 0 | 0 | 0 | 0 | 0 | 0.634 | 0.634 |
| Il10 | Mif | 10090.ENSMUSP00000016673 | 10090.ENSMUSP00000041149 | 0 | 0 | 0 | 0 | 0 | 0 | 0 | 0.637 | 0.637 |
| Il10 | Mapk3 | 10090.ENSMUSP00000016673 | 10090.ENSMUSP00000051619 | 0 | 0 | 0 | 0 | 0 | 0 | 0 | 0.675 | 0.675 |
| Il10 | Nos2 | 10090.ENSMUSP00000016673 | 10090.ENSMUSP00000018610 | 0 | 0 | 0 | 0 | 0 | 0 | 0 | 0.736 | 0.736 |
| Il10 | Ptgs2 | 10090.ENSMUSP00000016673 | 10090.ENSMUSP00000035065 | 0 | 0 | 0 | 0 | 0.071 | 0 | 0 | 0.83 | 0.835 |
| Il10 | Il2 | 10090.ENSMUSP00000016673 | 10090.ENSMUSP00000029275 | 0 | 0 | 0 | 0 | 0 | 0 | 0 | 0.95 | 0.951 |
| Il10 | Tlr4 | 10090.ENSMUSP00000016673 | 10090.ENSMUSP00000045770 | 0 | 0 | 0 | 0 | 0 | 0.538 | 0 | 0.92 | 0.961 |
| Il10 | Tnf | 10090.ENSMUSP00000016673 | 10090.ENSMUSP00000025263 | 0 | 0 | 0 | 0 | 0.149 | 0 | 0 | 0.962 | 0.967 |
| Il10 | Il6 | 10090.ENSMUSP00000016673 | 10090.ENSMUSP00000026845 | 0 | 0 | 0 | 0 | 0.157 | 0 | 0 | 0.973 | 0.977 |
| Il2 | Il4 | 10090.ENSMUSP00000029275 | 10090.ENSMUSP00000000889 | 0 | 0 | 0 | 0 | 0.062 | 0 | 0 | 0.954 | 0.955 |
| Il2 | Nos2 | 10090.ENSMUSP00000029275 | 10090.ENSMUSP00000018610 | 0 | 0 | 0 | 0 | 0 | 0 | 0 | 0.529 | 0.529 |
| Il2 | Tnf | 10090.ENSMUSP00000029275 | 10090.ENSMUSP00000025263 | 0 | 0 | 0 | 0 | 0.051 | 0 | 0 | 0.933 | 0.933 |
| Il2 | Il6 | 10090.ENSMUSP00000029275 | 10090.ENSMUSP00000026845 | 0 | 0 | 0 | 0 | 0 | 0 | 0 | 0.947 | 0.947 |
| Il2 | Mapk1 | 10090.ENSMUSP00000029275 | 10090.ENSMUSP00000065983 | 0 | 0 | 0 | 0 | 0 | 0 | 0 | 0.448 | 0.448 |
| Il2 | Ngf | 10090.ENSMUSP00000029275 | 10090.ENSMUSP00000102538 | 0 | 0 | 0 | 0 | 0 | 0 | 0 | 0.462 | 0.462 |
| Il2 | Mif | 10090.ENSMUSP00000029275 | 10090.ENSMUSP00000041149 | 0 | 0 | 0 | 0 | 0 | 0 | 0 | 0.482 | 0.482 |
| Il2 | Mapk3 | 10090.ENSMUSP00000029275 | 10090.ENSMUSP00000051619 | 0 | 0 | 0 | 0 | 0 | 0 | 0 | 0.585 | 0.585 |
| Il2 | Ptgs2 | 10090.ENSMUSP00000029275 | 10090.ENSMUSP00000035065 | 0 | 0 | 0 | 0 | 0 | 0 | 0 | 0.595 | 0.595 |
| Il2 | Tlr4 | 10090.ENSMUSP00000029275 | 10090.ENSMUSP00000045770 | 0 | 0 | 0 | 0 | 0 | 0 | 0 | 0.696 | 0.696 |
| Il4 | Mif | 10090.ENSMUSP00000000889 | 10090.ENSMUSP00000041149 | 0 | 0 | 0 | 0 | 0 | 0 | 0 | 0.522 | 0.522 |
| Il4 | Ngf | 10090.ENSMUSP00000000889 | 10090.ENSMUSP00000102538 | 0 | 0 | 0 | 0 | 0.062 | 0 | 0 | 0.537 | 0.547 |
| Il4 | Mapk3 | 10090.ENSMUSP00000000889 | 10090.ENSMUSP00000051619 | 0 | 0 | 0 | 0 | 0 | 0 | 0 | 0.585 | 0.585 |
| Il4 | Nos2 | 10090.ENSMUSP00000000889 | 10090.ENSMUSP00000018610 | 0 | 0 | 0 | 0 | 0.062 | 0 | 0 | 0.696 | 0.702 |
| Il4 | Ptgs2 | 10090.ENSMUSP00000000889 | 10090.ENSMUSP00000035065 | 0 | 0 | 0 | 0 | 0.062 | 0 | 0 | 0.779 | 0.784 |
| Il4 | Tlr4 | 10090.ENSMUSP00000000889 | 10090.ENSMUSP00000045770 | 0 | 0 | 0 | 0 | 0.062 | 0 | 0 | 0.817 | 0.821 |
| Il4 | Tnf | 10090.ENSMUSP00000000889 | 10090.ENSMUSP00000025263 | 0 | 0 | 0 | 0 | 0 | 0 | 0 | 0.903 | 0.903 |
| Il4 | Il6 | 10090.ENSMUSP00000000889 | 10090.ENSMUSP00000026845 | 0 | 0 | 0 | 0 | 0 | 0 | 0 | 0.958 | 0.958 |
| Il6 | Nqo1 | 10090.ENSMUSP00000026845 | 10090.ENSMUSP00000003947 | 0 | 0 | 0 | 0 | 0 | 0 | 0 | 0.543 | 0.543 |
| Il6 | Nos2 | 10090.ENSMUSP00000026845 | 10090.ENSMUSP00000018610 | 0 | 0 | 0 | 0 | 0.124 | 0 | 0 | 0.778 | 0.797 |
| Il6 | Tnf | 10090.ENSMUSP00000026845 | 10090.ENSMUSP00000025263 | 0 | 0 | 0 | 0 | 0.256 | 0 | 0 | 0.972 | 0.978 |
| Il6 | Slc1a5 | 10090.ENSMUSP00000026845 | 10090.ENSMUSP00000104136 | 0 | 0 | 0 | 0 | 0 | 0 | 0 | 0.457 | 0.457 |
| Il6 | Mif | 10090.ENSMUSP00000026845 | 10090.ENSMUSP00000041149 | 0 | 0 | 0 | 0 | 0 | 0 | 0 | 0.689 | 0.689 |
| Il6 | Ngf | 10090.ENSMUSP00000026845 | 10090.ENSMUSP00000102538 | 0 | 0 | 0 | 0 | 0.061 | 0 | 0 | 0.695 | 0.702 |
| Il6 | Lgals1 | 10090.ENSMUSP00000026845 | 10090.ENSMUSP00000086795 | 0 | 0 | 0 | 0 | 0.061 | 0 | 0.9 | 0.18 | 0.916 |
| Il6 | Tlr4 | 10090.ENSMUSP00000026845 | 10090.ENSMUSP00000045770 | 0 | 0 | 0 | 0 | 0.061 | 0 | 0 | 0.943 | 0.944 |
| Il6 | Ptgs2 | 10090.ENSMUSP00000026845 | 10090.ENSMUSP00000035065 | 0 | 0 | 0 | 0 | 0.442 | 0 | 0 | 0.912 | 0.949 |
| Il6 | Mapk1 | 10090.ENSMUSP00000026845 | 10090.ENSMUSP00000065983 | 0 | 0 | 0 | 0 | 0 | 0 | 0.9 | 0.55 | 0.953 |
| Il6 | Mapk3 | 10090.ENSMUSP00000026845 | 10090.ENSMUSP00000051619 | 0 | 0 | 0 | 0 | 0 | 0 | 0.9 | 0.783 | 0.977 |
| Mapk1 | Tnf | 10090.ENSMUSP00000065983 | 10090.ENSMUSP00000025263 | 0 | 0 | 0 | 0 | 0 | 0 | 0 | 0.72 | 0.72 |
| Mapk1 | Ptgs2 | 10090.ENSMUSP00000065983 | 10090.ENSMUSP00000035065 | 0 | 0 | 0 | 0 | 0 | 0.251 | 0 | 0.53 | 0.633 |
| Mapk1 | Tlr4 | 10090.ENSMUSP00000065983 | 10090.ENSMUSP00000045770 | 0 | 0 | 0 | 0 | 0 | 0.056 | 0 | 0.548 | 0.555 |
| Mapk1 | Mapk3 | 10090.ENSMUSP00000065983 | 10090.ENSMUSP00000051619 | 0 | 0 | 0.446 | 0.983 | 0 | 0.826 | 0.9 | 0.94 | 0.982 |
| Mapk1 | Ngf | 10090.ENSMUSP00000065983 | 10090.ENSMUSP00000102538 | 0 | 0 | 0 | 0 | 0 | 0 | 0 | 0.615 | 0.615 |
| Mapk3 | Nqo1 | 10090.ENSMUSP00000051619 | 10090.ENSMUSP00000003947 | 0 | 0 | 0 | 0 | 0 | 0 | 0 | 0.434 | 0.434 |
| Mapk3 | Nos2 | 10090.ENSMUSP00000051619 | 10090.ENSMUSP00000018610 | 0 | 0 | 0 | 0 | 0.05 | 0 | 0 | 0.584 | 0.588 |
| Mapk3 | Tnf | 10090.ENSMUSP00000051619 | 10090.ENSMUSP00000025263 | 0 | 0 | 0 | 0 | 0 | 0 | 0 | 0.773 | 0.773 |
| Mapk3 | Ptgs2 | 10090.ENSMUSP00000051619 | 10090.ENSMUSP00000035065 | 0 | 0 | 0 | 0 | 0 | 0.3 | 0 | 0.7 | 0.781 |
| Mapk3 | Mif | 10090.ENSMUSP00000051619 | 10090.ENSMUSP00000041149 | 0 | 0 | 0 | 0 | 0 | 0.207 | 0 | 0.398 | 0.502 |
| Mapk3 | Tlr4 | 10090.ENSMUSP00000051619 | 10090.ENSMUSP00000045770 | 0 | 0 | 0 | 0 | 0 | 0.056 | 0 | 0.699 | 0.704 |
| Mapk3 | Ngf | 10090.ENSMUSP00000051619 | 10090.ENSMUSP00000102538 | 0 | 0 | 0 | 0 | 0 | 0 | 0 | 0.686 | 0.686 |
| Mif | Tnf | 10090.ENSMUSP00000041149 | 10090.ENSMUSP00000025263 | 0 | 0 | 0 | 0 | 0 | 0 | 0 | 0.644 | 0.644 |
| Mif | Ptgs2 | 10090.ENSMUSP00000041149 | 10090.ENSMUSP00000035065 | 0 | 0 | 0 | 0 | 0 | 0 | 0 | 0.439 | 0.439 |
| Mif | Tlr4 | 10090.ENSMUSP00000041149 | 10090.ENSMUSP00000045770 | 0 | 0 | 0 | 0 | 0 | 0 | 0 | 0.52 | 0.52 |
| Nefh | Ngf | 10090.ENSMUSP00000091061 | 10090.ENSMUSP00000102538 | 0 | 0 | 0 | 0 | 0 | 0 | 0 | 0.643 | 0.643 |
| Ngf | Tnf | 10090.ENSMUSP00000102538 | 10090.ENSMUSP00000025263 | 0 | 0 | 0 | 0 | 0 | 0 | 0 | 0.68 | 0.68 |
| Ngf | Ptgs2 | 10090.ENSMUSP00000102538 | 10090.ENSMUSP00000035065 | 0 | 0 | 0 | 0 | 0.095 | 0 | 0 | 0.503 | 0.531 |
| Ngf | Tlr4 | 10090.ENSMUSP00000102538 | 10090.ENSMUSP00000045770 | 0 | 0 | 0 | 0 | 0 | 0.096 | 0 | 0.382 | 0.417 |
| Nos2 | Tlr4 | 10090.ENSMUSP00000018610 | 10090.ENSMUSP00000045770 | 0.041 | 0 | 0 | 0 | 0.062 | 0 | 0 | 0.812 | 0.816 |
| Nos2 | Tnf | 10090.ENSMUSP00000018610 | 10090.ENSMUSP00000025263 | 0 | 0 | 0 | 0 | 0.088 | 0 | 0 | 0.904 | 0.909 |
| Nos2 | Ptgs2 | 10090.ENSMUSP00000018610 | 10090.ENSMUSP00000035065 | 0 | 0 | 0 | 0 | 0.107 | 0.545 | 0 | 0.847 | 0.932 |
| Nqo1 | Ptgs2 | 10090.ENSMUSP00000003947 | 10090.ENSMUSP00000035065 | 0 | 0 | 0 | 0 | 0 | 0 | 0 | 0.524 | 0.524 |
| Nqo1 | Tnf | 10090.ENSMUSP00000003947 | 10090.ENSMUSP00000025263 | 0 | 0 | 0 | 0 | 0 | 0 | 0 | 0.527 | 0.527 |
| Ptgs2 | Tnf | 10090.ENSMUSP00000035065 | 10090.ENSMUSP00000025263 | 0 | 0 | 0 | 0 | 0.117 | 0 | 0 | 0.915 | 0.922 |
| Ptgs2 | Tlr4 | 10090.ENSMUSP00000035065 | 10090.ENSMUSP00000045770 | 0 | 0 | 0 | 0 | 0.062 | 0 | 0 | 0.752 | 0.757 |
| Tlr4 | Tnf | 10090.ENSMUSP00000045770 | 10090.ENSMUSP00000025263 | 0 | 0 | 0 | 0 | 0.072 | 0.05 | 0 | 0.931 | 0.935 |

**Table S4. Information of GO enrichment analysis**

| **Category** | **Term** | **Description** | **LogP** | **Log(q-value)** | **Count** | **Ratio** |
| --- | --- | --- | --- | --- | --- | --- |
| GO Biological Processes | GO:0070997 | neuron death | -35.418 | -31.221 | 35 | 0.398 |
| GO Biological Processes | GO:1901214 | regulation of neuron death | -33.897 | -30.002 | 33 | 0.375 |
| GO Biological Processes | GO:0001505 | regulation of neurotransmitter levels | -32.330 | -28.611 | 32 | 0.364 |
| GO Biological Processes | GO:0014070 | response to organic cyclic compound | -26.951 | -23.356 | 35 | 0.398 |
| GO Biological Processes | GO:0007610 | behavior | -26.716 | -23.219 | 35 | 0.398 |
| GO Biological Processes | GO:0050890 | cognition | -26.278 | -22.860 | 27 | 0.307 |
| GO Biological Processes | GO:0051402 | neuron apoptotic process | -26.196 | -22.845 | 26 | 0.295 |
| GO Biological Processes | GO:0043523 | regulation of neuron apoptotic process | -25.893 | -22.599 | 25 | 0.284 |
| GO Biological Processes | GO:0010942 | positive regulation of cell death | -24.985 | -21.743 | 33 | 0.375 |
| GO Biological Processes | GO:0007611 | learning or memory | -24.651 | -21.455 | 25 | 0.284 |
| GO Cellular Components | GO:0043025 | neuronal cell body | -24.832 | -21.549 | 33 | 0.375 |
| GO Cellular Components | GO:0030424 | axon | -23.477 | -20.495 | 32 | 0.364 |
| GO Cellular Components | GO:0033267 | axon part | -19.069 | -16.263 | 24 | 0.273 |
| GO Cellular Components | GO:0150034 | distal axon | -18.273 | -15.592 | 22 | 0.250 |
| GO Cellular Components | GO:0098793 | presynapse | -17.733 | -15.149 | 25 | 0.284 |
| GO Cellular Components | GO:0030425 | dendrite | -15.401 | -12.923 | 25 | 0.284 |
| GO Cellular Components | GO:0097447 | dendritic tree | -15.361 | -12.923 | 25 | 0.284 |
| GO Cellular Components | GO:0043679 | axon terminus | -11.735 | -9.355 | 13 | 0.148 |
| GO Cellular Components | GO:0044306 | neuron projection terminus | -11.139 | -8.853 | 13 | 0.148 |
| GO Cellular Components | GO:0098889 | intrinsic component of presynaptic membrane | -11.136 | -8.853 | 11 | 0.125 |
| GO Molecular Functions | GO:0019901 | protein kinase binding | -11.350 | -7.690 | 21 | 0.239 |
| GO Molecular Functions | GO:0070405 | ammonium ion binding | -7.622 | -4.334 | 7 | 0.080 |
| GO Molecular Functions | GO:0030594 | neurotransmitter receptor activity | -7.517 | -4.334 | 8 | 0.091 |
| GO Molecular Functions | GO:0005126 | cytokine receptor binding | -7.012 | -3.954 | 11 | 0.125 |
| GO Molecular Functions | GO:0097200 | cysteine-type endopeptidase activity involved in execution phase of apoptosis | -6.825 | -3.864 | 4 | 0.045 |
| GO Molecular Functions | GO:0031072 | heat shock protein binding | -6.681 | -3.852 | 8 | 0.091 |
| GO Molecular Functions | GO:0097153 | cysteine-type endopeptidase activity involved in apoptotic process | -6.667 | -3.852 | 4 | 0.045 |
| GO Molecular Functions | GO:0070851 | growth factor receptor binding | -6.569 | -3.812 | 8 | 0.091 |
| GO Molecular Functions | GO:0019902 | phosphatase binding | -6.295 | -3.589 | 9 | 0.102 |
| GO Molecular Functions | GO:0019903 | protein phosphatase binding | -6.025 | -3.365 | 8 | 0.091 |

**Table S5. Information of KEGG enrichment analysis**

| **Category** | **Term** | **Description** | **LogP** | **Log(q-value)** | **Count** | **Ratio** |
| --- | --- | --- | --- | --- | --- | --- |
| KEGG Pathway | mmu05210 | Colorectal cancer | -12.617 | -10.016 | 10 | 0.114 |
| KEGG Pathway | mmu04210 | Apoptosis | -12.140 | -9.840 | 12 | 0.136 |
| KEGG Pathway | mmu05200 | Pathways in cancer | -11.557 | -9.477 | 17 | 0.193 |
| KEGG Pathway | mmu05014 | Amyotrophic lateral sclerosis | -11.485 | -9.477 | 9 | 0.102 |
| KEGG Pathway | mmu04010 | MAPK signaling pathway | -11.203 | -9.282 | 14 | 0.159 |
| KEGG Pathway | mmu01522 | Endocrine resistance | -11.143 | -9.282 | 10 | 0.114 |
| KEGG Pathway | mmu05140 | Leishmaniasis | -10.731 | -8.975 | 9 | 0.102 |
| KEGG Pathway | mmu05152 | Tuberculosis | -10.582 | -8.856 | 12 | 0.136 |
| KEGG Pathway | mmu05145 | Toxoplasmosis | -10.404 | -8.736 | 10 | 0.114 |
| KEGG Pathway | mmu05161 | Hepatitis B | -10.382 | -8.736 | 11 | 0.125 |
| KEGG Pathway | mmu04024 | cAMP signaling pathway | -10.243 | -8.642 | 12 | 0.136 |
| KEGG Pathway | mmu05206 | MicroRNAs in cancer | -9.649 | -8.109 | 13 | 0.148 |
| KEGG Pathway | mmu05215 | Prostate cancer | -9.328 | -7.857 | 9 | 0.102 |
| KEGG Pathway | mmu04080 | Neuroactive ligand-receptor interaction | -9.285 | -7.830 | 13 | 0.148 |
| KEGG Pathway | mmu04933 | AGE-RAGE signaling pathway in diabetic complications | -9.176 | -7.751 | 9 | 0.102 |
| KEGG Pathway | mmu05142 | Chagas disease | -9.176 | -7.751 | 9 | 0.102 |
| KEGG Pathway | mmu05010 | Alzheimer disease | -8.905 | -7.542 | 11 | 0.125 |
| KEGG Pathway | mmu04725 | Cholinergic synapse | -8.890 | -7.542 | 9 | 0.102 |
| KEGG Pathway | mmu04510 | Focal adhesion | -8.861 | -7.527 | 11 | 0.125 |
| KEGG Pathway | mmu05030 | Cocaine addiction | -8.758 | -7.445 | 7 | 0.080 |

**Table S6. Top 10 hub targets calculated by MCC method**

| **Rank** | **Name** | **Score** |
| --- | --- | --- |
| 1 | Akt1 | 1.81E+12 |
| 2 | Tnf | 1.81E+12 |
| 3 | Ptgs2 | 1.81E+12 |
| 4 | Casp3 | 1.81E+12 |
| 5 | Igf1 | 1.81E+12 |
| 6 | Sirt1 | 1.81E+12 |
| 7 | Jun | 1.81E+12 |
| 8 | Cat | 1.80E+12 |
| 9 | Il10 | 1.79E+12 |
| 10 | Bcl2l1 | 1.78812E+12 |
